# Open Supracondylar Humerus Fractures in Children

**DOI:** 10.7759/cureus.13903

**Published:** 2021-03-15

**Authors:** Tommy Pan, Matthew R Widner, Michael M Chau, William L Hennrikus

**Affiliations:** 1 Department of Orthopaedics and Rehabilitation, Penn State College of Medicine, Hershey, USA; 2 Department of Orthopaedic Surgery, University of Minnesota Twin Cities, Minneapolis, USA; 3 Department of Orthopaedics and Rehabilitation, Penn State Health Milton S. Hershey Medical Center, Hershey, USA

**Keywords:** supracondylar, fracture, open, gartland, flynn, pediatric

## Abstract

Purpose: Supracondylar humerus (SCH) fractures are the most common elbow fracture in children; however, they rarely occur as open injuries. Open fractures are associated with higher rates of infection, neurovascular injury, compartment syndrome, and nonunion. The purpose of this study was to evaluate the treatment and outcomes of open SCH fractures in children.

Methods: Between 2008 and 2015, four children (1%) had open injuries among 420 treated for SCH fractures at a single center. The mean patient age was six years (range, four to eight years). Two patients had Gustilo-Anderson grade 1 open fractures and two had grade 2 fractures. Tetanus immunization was up-to-date in all. First dose of intravenous antibiotics was given on average 3hr 7min after onset of injury (range, 1hr 38min to 8hr 15min). Time from injury to irrigation and debridement (I&D) and closed reduction and percutaneous pinning (CRPP) was on average 8hr 16min (range, 4hr 19min to 13hr 15min). All patients received 24-hour intravenous antibiotics. Pins were removed at four weeks and bony union occurred by six weeks.

Results: After an average follow-up period of 12 months (range, 6 to 22 months), there were no infections, neurovascular deficits, compartment syndromes, cubitus varus deformities, or range of motion losses. All outcomes were excellent according to the Flynn criteria. Due to the unstable nature of open SCH fractures, a medial pin was used in all four cases. No loss of reduction or ulnar nerve injury occurred.

Conclusion: Open injuries occur in approximately 1% of all SCH fractures in children. The authors recommend urgent intravenous antibiotics, I&D, and CRPP involving a medial pin to treat open SCH fractures. Excellent outcomes based on the Flynn criteria were obtained in four cases.

## Introduction

Supracondylar humerus (SCH) fractures are the most common elbow fractures in children [1]. They account for approximately 3% of all childhood fractures [2] and are the most common pediatric fracture requiring surgery [3]. Previous authors have published rates of open SCH fractures ranging between 0.5% and 4.5% [4-10]. Excluding one outlying study [9], the overall rate of open SCH fractures was approximately 1% (n = 3300). Open injuries are associated with greater fracture displacement and a higher rate of neurovascular injury [4-11]. Open fractures typically also increase the risk of infection, compartment syndrome, and nonunion [12].

Understanding the management of open SCH fractures in children could reduce perioperative complications, and optimize long-term results for patients. The aim of this study was to evaluate the treatment and outcomes of open SCH fractures in children.

## Materials and methods

Institutional review board approval was obtained for this retrospective case series. The pediatric fracture database was retrieved at the authors’ Level I Pediatric Trauma Center from 2008 to 2015. Electronic medical records were systematically reviewed to identify patients who satisfy the following inclusion criteria: <13-years-old, open fracture, SCH fracture, and >6 months of follow-up. Open fractures were graded using the Gustilo-Anderson classification (Table 1) [13] and SCH fractures were graded retrospectively on imaging using the modified Gartland classification (Table 2) [14]. Grading was performed by the senior author (WH). For each case, the following data were collected: age at injury, gender, immunization history, Gustilo-Anderson type, modified Gartland type, time from injury to an outside hospital followed by transfer to the authors’ emergency department, time from injury to the first dose of intravenous antibiotics, time from injury to irrigation and debridement (I&D), time from injury to cessation of immobilization, length of follow-up, and complications (i.e., infection, neurovascular deficit, compartment syndrome, and nonunion). The Flynn criteria was used to grade functional and cosmetic outcomes at the last follow-up [15] (Table 3). Two surgeons treated the patients. Both surgeons had more than 25 years of experience.

**Table 1:** Modified Gartland Classification for Supracondylar Humerus Fractures

Types	Description
I	Nondisplaced
II	Displaced with partially intact posterior cortex
III	Displaced without any cortical continuity
IV	Type III plus circumferential periosteal disruption causing multidirectional instability

**Table 2:** Gustilo-Anderson Classification for Open Fractures

Type	Description
I	Wound <1 cm, minimal soft tissue damage and contamination, simple fracture pattern
II	Wound >1 cm, moderate soft tissue damage, contamination, and comminution
III	Wound usually >10 cm, extensive soft tissue damage and contamination, segmental fracture or severe comminution. IIIA: Adequate soft tissue remaining to cover bone. IIIB: Requires local or distant soft tissue flap to cover bone. IIIC: Any open fracture with arterial injury requiring repair.

**Table 3:** Flynn Criteria for Outcomes of Supracondylar Humerus Fractures ^a^The lower of the two ratings is the overall rating.

Outcome^a^	Cosmetic factor: carrying-angle loss	Functional factor: range of motion loss
Excellent	0-5°	0-5°
Good	5-10°	5-10°
Fair	10-15°	10-15°
Poor	>15°	>15°

Perioperative management

All patients who presented with an open fracture received intravenous antibiotics in the emergency room (ER) as soon as possible. Once a SCH fracture was diagnosed, a sterile dressing was placed over the wound and a posterior splint was applied with the elbow flexed at approximately 25° to provide provisional stabilization. All patients were taken to the next available operating room for I&D and open assisted reduction and pinning. All patients received intravenous antibiotics for 24 hours. Tetanus immunization status was verified in all patients.

Surgical technique

Fracture type was confirmed intraoperatively by fluoroscopy. I&D was performed by extending the traumatic anterior wound approximately 1 cm obliquely in each direction to address the zone of injury and expose the fracture ends. Both bone ends were delivered through the wound. Debridement of contaminated bone and soft tissue edges was performed and the wound was irrigated copiously with normal saline. Open assisted reduction was then performed using the partly intact periosteum as a hinge. Extension-type fractures were reduced by traction, translation, and flexion at the elbow. Flexion-type fractures were reduced by traction and elbow extension. Reduction was confirmed by fluoroscopy. Fixation was performed using two laterally based 2 mm smooth Steinmann pins followed by an additional smooth pin on the medial side to maximize stability. When placing the medial pin, the elbow was extended to release tension on the soft tissues, the ulnar nerve was identified and avoided through a small incision over the medial condyle, and the pin was placed directly on bone. Pin positioning was confirmed by fluoroscopy. The extended part of the wound was closed using absorbable sutures and the traumatic open wound was sterilely dressed and allowed to heal by secondary intention as per our institution’s traditional management preference. The pins were left percutaneous and removed in four weeks in clinic.

## Results

A total of 4 out of 420 children (1%) with SCH fractures had open injuries over an 8-year period (Table 4). Mean patient age was six years (range, four to eight years). There were three boys and one girl. One fracture was Gustilo-Anderson type II and Garland extension-type III; two fractures were Gustilo-Anderson type I and Gartland extension-type III; one fracture was Gustilo-Anderson type II and Gartland flexion-type II. All extension-type SCH fractures occurred in the nondominant arm of male patients caused by fall from height onto an outstretched arm. The flexion-type SCH fracture occurred in the dominant arm of a female patient as a result of a direct blow to the elbow. Three patients were initially stabilized at an outside hospital and transferred to our facility for definitive treatment.

First dose of intravenous antibiotics was given in the ER on average 3hr 7min after onset of injury (range, 1hr 38min to 8hr 15min). The patient who received late intravenous antibiotics was delayed due to initial treatment at an outside hospital. Time from injury to I&D and open assisted reduction was on average 8hr 16min (range, 4hr 19min to 13hr 15min). There was a delay to surgery in two patients due to transfer from an outside hospital and operating room availability. All patients received 24 hours of intravenous antibiotics. All of the patients’ pins were removed at four weeks to prevent pin infections. The elbow was immobilized for an additional two weeks in a splint to prevent fracture displacement. After an average follow-up period of 12 months (range, 6 to 22 months), there were no infections, neurovascular deficits, compartment syndromes, cubitus varus deformities, or range of motion losses. All outcomes were excellent according to the Flynn criteria (Table 3).

**Table 4:** Open Supracondylar Humerus Fractures in Four Children ^a^A total of 420 children with supracondylar humerus fractures were treated over an eight-year period. ^b^Patient was transferred to our emergency department (ED) from an outside hospital (OSH).

Characteristic^a^	Patient 1	Patient 2	Patient 3	Patient 4	Average
Age	4yr	6yr	7yr	8yr	6yr
Gender	Male	Male	Male	Female	-
Gartland type	III (extension)	III (extension)	III (extension)	II (flexion)	-
Gustilo-Anderson type	I	I	II	II	-
Time: injury to OSH^b^	1hr 2min	0hr 40min	-	0hr 58min	0hr 53min
Time: injury to ED	5hr 34min	3hr 5min	2hr 10min	3hr 9min	3hr 30min
Time: injury to first antibiotics	8hr 15min	1hr 38min	2hr 35min	2hr 0min	3hr 7min
Time: injury to surgery	11hr 0min	4hr 31min	4hr 19min	13hr 15min	8hr 16min
Length of follow-up	22mo	6mo	13mo	6mo	12mo
Flynn criteria	Excellent	Excellent	Excellent	Excellent	-

## Discussion

Open SCH fractures are rare but potentially troublesome injuries in children and specific literature is limited [9,16]. The current study evaluates treatment and outcomes of open SCH fractures in children with intention to guide future management of these injuries.

A primary goal of treating any open fracture is to prevent infection [12,17]. Gustilo and Anderson showed a decrease in infection rate in open fractures when antibiotics were started within three hours of the injury compared to three or more hours [18]. In the current study, the average time from onset of injury to first dose of intravenous antibiotics was 3hr 7min and to I&D was 8hr 16min. Despite only two patients receiving I&D within six hours for open fractures [13], there was no soft tissue infection or osteomyelitis. Stewart et al. have noted that abundant blood supply about the elbow contributes to a low infection rate [12]. Recent studies have also suggested that the timing of I&D for low-grade open fractures can be delayed up to 24 hours without increasing the risk of morbidity [17]. However, traditional teaching holds that the most effective intervention for preventing infection is urgent intravenous antibiotics combined with I&D in the first available operating room [13,18]. Two studies demonstrated no difference in infection rate with a short versus long duration of antibiotics [19,20].

Open injury is a commonly accepted indication for using open reduction to treat SCH fractures [21,22]. Although good outcomes with open techniques have been described [22], open reduction of a child’s small elbow requires a large incision to apply clamps to open the reduced fracture. Therefore, open reduction increases the risk of elbow stiffness due to the large surgical exposure [6]. A recent study by Chang et al. demonstrated that open SCH fractures that underwent open reduction had decreased range of motion at final follow-up compared to those that had an I&D followed by closed reduction [23]. The current study reports excellent outcomes and no loss of motion with an I&D via a smaller surgical exposure followed by open assisted reduction with percutaneous pinning. Ozkul et al. also described favorable results using a similar technique to treat open SCH fractures [9].

The use of lateral pins is acceptable for fixing most SCH fractures [17]. Medial pin placement carries a risk of iatrogenic ulnar nerve injury [24,25]. In the case of an open Gartland type 3 SCH fracture, a medial pin enhances torsional stability [6,25,26]. In the current study, no patient lost reduction and no case of cubitus varus occurred. There was also no ulnar nerve injury. Intraoperative methods used in the current study to protect the ulnar nerve included placing lateral pins before the medial pin, elbow extension, and direct visualization using a mini open approach to place the medial pin [27].

All four patients in the current study had excellent outcomes according to the Flynn criteria. Trionfo et al. reported a small series of children with open SCH fractures treated with I&D within 12 hours of injury followed by open reduction and crossed percutaneous pinning [17]. All outcomes were excellent [17]. Özkul et al reported another series of children with open SCH fractures treated with I&D within eight hours of injury followed by closed reduction and crossed percutaneous pinning [9]. All patients had excellent or good outcomes and one patient had a pin tract infection [9]. Long-term results of open SCH fractures are comparably favorable to their closed counterparts when treated appropriately [25].

Similar to closed SCH fractures in terms of epidemiology, open SCH fractures in the current study occurred at a peak age of four years (range, four to eight years) [3,4,8], usually involved the nondominant arm (3:1) [4,8], and were more common in boys (3:1) [4].

Due to the paucity of open pediatric humerus fractures, there is no consensus on the effect of surgical timing, closed versus open reduction, and positioning of fixation pins. Few studies in the literature have specifically addressed open SCH fractures in children [9,16]. Therefore, these injuries are treated similarly to their closed counterparts with the addition of basic open fracture principles such as intravenous antibiotics as soon as possible, tetanus prophylaxis as needed, irrigation and debridement (I&D) and internal fixation [2,4-7,9-11,16,21,22]. As demonstrated in the current study, pediatric open fractures typically have better outcomes when compared to adults due to a thickened and highly vascularized periosteum leading to increased healing rates, quicker time to union, and more robust bone regeneration [28].

To summarize, the current study demonstrates that a judicious I&D followed by an open assisted reduction with percutaneous pinning is safe and effective when treating these uncommon injuries. In addition, a small medial incision was done to safely place the medial pin as noted by prior authors [27].

The limitations of the current study are its retrospective design and small number of open SCH fractures.

## Conclusions

Open injuries occur in approximately 1% of all SCH fractures in children. The authors recommend urgent intravenous antibiotics and I&D. In addition, open assisted reduction and pinning via small incision rather than a formal open reduction via large incisions resulted in excellent elbow range of motion without infection. To address the unstable nature of these injuries, a medial pin utilizing a small incision to protect the ulnar nerve is recommended. Excellent outcomes based on the Flynn criteria were obtained in four cases.
